# The value of squamous cell carcinoma antigen (SCCa) to determine the lymph nodal metastasis in cervical cancer: A meta-analysis and literature review

**DOI:** 10.1371/journal.pone.0186165

**Published:** 2017-12-11

**Authors:** Ziqi Zhou, Wenbo Li, Fuquan Zhang, Ke Hu

**Affiliations:** Department of radiation oncology, Peking Union Medical College Hospital. Chinese Academy of Medical Sciences and Peking Union Medical College, Beijing, People’s Republic of China; Universidade Estadual de Maringa, BRAZIL

## Abstract

**Background:**

The diagnostic power of CT or MRI on the lymph node status was limited. Supplement measurements were needed to assist the diagnosis of lymph node metastasis. The SCCa was reported to be close related to lymph node status. But currently the clinical value of serum SCCa measurement in lymph node status has not been clearly defined. This meta-analysis was to investigate this topic on a large scale.

**Method:**

Searching the Pubmed, Embase, Cochrane library, CNKI and Wanfang database for SCC-Ag/SCCA/SCC-antigen and cervical cancer/tumor/carcinoma/neoplasm published in any language from Jan 1 1990 to Aug 1 2017. QUADAS (quality assessment of diagnostic accuracy studies) was used to evaluate the quality of the articles. An eligible set of data should include true positive, true negative, false positive and false negative number. Every set of data was extracted and analyzed by STATA 14.0. The forest plot and bivariate boxplot were utilized to evaluate the heterogeneity. The funnel graph was used to test the publication bias. The SROC curve was draw via random effect model and HSROC model.

**Result:**

17 sets of data and 3985 patients were included for the diagnostic meta-analysis. There was heterogeneity, which was partially from SCCa cut-off value. The pooled sensitivity was 0.70 and specificity was 0.63. AUC was 0.73. Eight articles provided the relative risk value of lymphatic metastasis when SCCa increased. The relative risk of lymph node metastasis increased ranging from 2.3–40 as with different SCCa cut off value.

**Conclusion:**

The diagnostic value of SCCa for lymph nodal metastasis was medium and it was strongly related to lymph node status. Thus SCCa could assist imaging tests to detect lymph node metastasis. Besides, it was correlated with para-aortic lymph node metastasis.

## Introduction

Cervical cancer is a major gynecological cancer, which involves uncontrolled cell division and tissue invasiveness of the female uterine cervix. It is the second major malignant tumor in women[1]. Half a million women are diagnosed as having cervical cancer each year in the world, leading to 9% of the deaths in women[2].

Lymph node metastasis is one of the most important clinical parameters in the treatment determination and prognosis for carcinoma of the cervix. Lymph node metastasis is the main factor affecting the 5-year overall survival rate of cervical cancer patients [3, 4]. In early stage cervical carcinoma, the 5-year overall survival rate of lymph node positive patients was reported to be around 50%, while that of non-lymph node metastasis was more than 90%[5]. The prognosis of cervical carcinoma with common iliac lymph node metastasis was even poorer, with the 5-year overall survival rate of 25%-47.8%[5, 6]. Therefore, before treating patients, it is necessary to judge the lymphatic status of the patients and evaluate the lymph node metastasis risk.

Preoperative assessment for lymph node metastasis usually employs CT or MRI. But it has been reported that imaging analysis using CT or MRI has a high false negative rate for lymph node metastasis[7]. The clinical value of CT is limited by the low positive predictive value (75%) [3]. Microscopic metastasis accounts for about 26% of metastases[8]. It is unlikely that this microscopic metastasis is to be detected by CT scan. Thus it is necessary to find some indicators which strongly associated with lymph node metastasis to assist CT or MRI.

Squamous cell carcinoma antigen (SCCa), which was first described by Kato and Torigoe[9], is the most widely used and most reliable tumor marker for squamous cell carcinoma[10]. There are abundant reports currently in the literature regarding the importance of the pre-therapeutic SCCa level in the serum as a prediction factor for lymph node metastasis. Whether SCCa could serve as a reliable prediction factor for lymph node metastasis was still remained controversial so far. Thus, we went over those studies about the experience of SCCa elevation in relation to lymph node status and then meta-analyzed them on a large scale, in order to determine whether a serum marker squamous cell carcinoma antigen can predict the presence of nodal metastasis.

## Material and method

### Inclusion and exclusion criteria

Two independent reviewers screened all identified articles, and compared selected studies. Disagreement was resolved by consensus. Studies were included if they fulfilled the following criteria:(1) the cases which were diagnosed with cervical carcinoma (2) in the case group the lymph node metastasis was confirmed by pathology (3) the SCCa test was certain and could be repeated; (4) the data was presented as an entire set which include true positive, true negative, false positive and false negative number (5) The RR/OR/HR, the number of cases, FIGO stage, must be also provided (or data to calculate these). When there were multiple publications from the same population, the study that included the most recently updated data was included. Excluded if 1) The set of data was not complete (eg. only TP was available, but TN, FP and FN were missing.) 2) Lymph nodal metastasis was not confirmed by pathology.

### Search strategy

Eligible studies were identified by searching the Pubmed, Embase, Cochrane library,CNKI and Wanfang database for pertinent articles published in any language from Jan 1 1990 to Aug 1 2017 and by manually searching the reference list of the computer retrieved publications. For the computer searches we used the following MeSH terms or text words: A or a combined with B or b. A was defined as squamous cell carcinoma antigen, SCC-Ag, SCCA, SCC-antigen. B was defined as cervical cancer/tumor/carcinoma/neoplasm. We selected articles related with the SCCa and cervical cancer by title. Then we read the abstract to pick out articles associated with SCCa and lymph node status. Then we read the full text and pick out articles which were highly related to our topic Studies were included in the meta-analyses if they presented data on the association between SCCa and lymph nodal metastasis in cervical cancer patients. To be included, studies had to present the entire set of TP, TN, FP and FN, or sufficient data to permit their calculations.

### Data abstraction

Two authors independently completed the data extraction. Any disagreements were resolved by discussion and end with consensus. The data which we abstracted from the publications included the first author’s name, year of publication, FIGO stages, number of patients, number of patients who have the laparoscopic biopsy laparoscopic biopsy or lymphadenectomy, cut-off value of SCCa, sensitivity, specificity, negative predictive value, positive predictive value, true negativity, false negativity, true positivity was abstract for diagnostic meta analysis. The HR/RR/ORs (95%CI) were extracted as well.

### Quality evaluation

Two authors independently completed the quality evaluation using QUADAS (quality assessment of diagnostic accuracy studies) which included 14 items. Each item could be judged as yes (recorded as 2), no(recorded as 0) or not clearly(recorded as 1).

### Heterogeneity evaluation

We draw the forest plots and bivariate boxplot on Stata 14.0. Evaluation of heterogeneity was based on the I^2^and the dots on the boxplot. I^2^>50% indicating significant heterogeneity. If there was heterogeneity between studies, we would drill down the factors accounting for that and utilize the random effect model to meta-analyze.

### Risk of bias

We evaluate the bias according to the Agency for Healthcare Rearch and Quality(AHRQ) evidence based practice center(EPC) methods guide. The possibility of publication bias was assessed by visual inspection of a funnel plot by Stata 14.0. The P>0.1 was considered as no publication bias.

### Statistic analysis

Stata 14.0 was utilized to analyze the data. *P<*0.05 was considered as statistically significant.

### Analysis of the results

If there was no heterogeneity, the fixed effect model was to be used. If there was heterogeneity, we used the random effect model to analyze the diagnostic value of SCCa via pooled sensitivity, specificity, negative predictive value (NPV), positive predictive value(PPV) etc. Then the SROC curve was drawn and the AUC was obtained. For SROC curve, the diagnostic value was better when the AUC value was closer to 1.When the AUC was between 0.5–0.7, the diagnostic value was considered low. When the AUC was between 0.7–0.9, the diagnostic value was considered medium. When the AUC was>0.9, the diagnostic value was considered high. Additionally if heterogeneity existed, subgroup analyses would be done.

## Result

**Fig 1:**
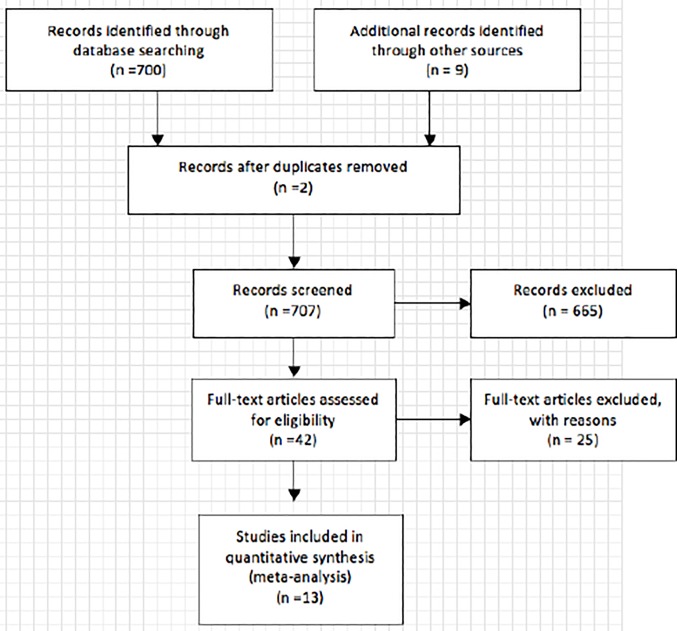
The PRISMA flow diagram of literature screening. 13 articles meet the inclusion and exclusion requirement eventually and were eligible for the meta-analysis.

We initially retrieved a total number of 709 articles from the database and the bibliography of the primary studies, in which 564 articles were excluded based on titles in the first screening, leaving 145 articles for abstract review. 42 articles mentioned the predictive value of SCCa on lymph node metastasis in which 13 articles provide 17 sets of data. The flow chart was draw in Fig 1.

### Literature review regarding the prediction value of SCCa on lymphatic node metastasis

In 1995 Gaarenstroom [11]investigated the Clinical value of pretreatment SCCa in 78 patients of stage I-IV. The clinical performance of SCCa (>1.5ng/ml) predicted lymph node metastases with the sensitivity and specificity of 55.5% and 58.3%. After adjusting for tumor stage and size, it was found not statistically significant, which means SCCa(>1.5ng/ml) was not predictive for the presence of lymph node metastases.

In 2000 Gaarenstroom[12] study the Clinical value of pretreatment SCCa in 63 early stage squamous cell cervical cancer patients (stage I-IIA). The sensitivity and specificity of SCC-Ag (>1.5ng/ml) for the presence of lymph node metastases was 73% and 56%, respectively. Its conclusion was that SCCa was insufficiently reliable for identifying patients at risk of the presence of lymph node metastases.

In 1997, Bolger [3]studied 220 patients with surgically treated early-stage cervical carcinoma and found the positive predictive value of SCCa for lymph node metastases at 2, 4, and 8.6ng/ml is 51.4, 70.0, and 100%. The sensitivity is 58.1, 45.2, and 22.6%, respectively. SCCa exceeding 8.6ng/ml is highly predictive of lymph nodal metastases with the positive and negative predictive value of 100% and 83.9%respectively.

Duk[13] studied 653 women diagnosed with squamous cervical cancer. It was found that the elevated pretreatment SCCa correlated with the number of affiliated lymph nodes. For stage IB and IIA patients, SCC exceeding1.9ng/ml predict 4 times the possibility of more than one positive lymph node (24% versus 6%, *P<*0.05). The sensitivity and specificity of SCCa>1.9ng/ml was 64.6% and 68.4%. By exclusion of patients with >4cm tumor size or grade III and IV, the sensitivity and specificity was 73.3% and 73.3%.

Feng[14] investigated the risk factors of node-positive cervical carcinoma in 205 patients and found SCCa>4ng/ml increased the risk of nodal metastasis by 4.2 folds (OR = 4.212, *P*<0.05). The obturator and obturator fossae lymph nodes were the most frequently involved, with a rate of 48.0%. Moreover, 60.0% node-positive patients had multiple sites lymph node metastases.

Lin[15] studied 284 patients with stage IB and IIA cervical cancer undergoing radical hysterectomy. It was found that when the cutoff value of SCCa for lymph node metastasis was set at 8 mg/l, the sensitivity was 35.7% and the specificity was 95.2%. 85.8% patients with a level <8ng/ml were node negative and the positive predictive value was 64.5%. Lin concluded that for predicting nodal metastasis preoperatively, SCCa levels greater than 8ng/ml can be considered a high-risk zone for nodal metastasis. Preoperative serum SCCa is not sensitive enough to screen for nodal disease in patients with early stage squamous cell. But an elevated level might provide extra information to the clinicians and to alert them to the possibility of nodal metastasis.

Luan[16] investigated the relationship between SCCa and lymph node metastases on 1195 patients with cervical squamous cell carcinoma. It was found that the areas under ROC curve for pelvic lymph node metastasis were 0.71. From the ROC curve the best cutoff value of SCCa for lymph node metastasis was 2.15ng/ml. The corresponding sensitivity and specificity was 69.7% and 66.4%. SCCa level above 2.15ng/ml increased the risk of pelvic lymph nodal metastasis by 3.78 folds. Lin concluded that squamous cell carcinoma antigen is correlated with lymph node metastasis. The best threshold value of pretreatment serum SCCa for predicting early postoperative cervical lymph node metastasis was 2.15ng/ml.

Huang[17] investigated the risk factors and prognosis of IB-IIB cervical carcinoma with common iliac lymph node metastasis in 960 patients who received radical hysterectomy and bilateral pelvic lymphadenectomy. SCCa >4 ng/ml before treatment was found to be the independent risk factors in the common iliac lymph node metastasis. The common iliac lymph node metastasis risk of patients with pretreatment SCCa > 4ng/ml was 2.3 times that of patients with SCCa<4 ng/ml. By using the cut-off value 4ng/ml in patients with stage IB-IIB, the negative predictive value for common iliac node metastasis was 96%. Huang suggested that rapid pathological section should be performed for suspected metastatic lymph node of patients with SCCa> 4ng/ml. If the results were positive, biopsy taking or peritoneal para-aortic lymph node removal was necessary, which avoided unnecessary lymph node removal or misdiagnosis of lymph node metastasis

Kim[18] explored the predicting value of SCCA on lymph node metastases in 104 patients with early stage (IB-IIA) cervical carcinoma. It was found that patients with positive nodal involvement has the SCCa 3.9ng/ml while the negative node has 1.1ng/ml. Employing the cutoff value of 2.0ng/mL for SCCA, the sensitivity and specificity of SCCA levels for nodal involvement were 63.2% and 52.9%, respectively.

Pan[19] studied 82 patients from IA-IVB and found the positive node patients had higher SCC than the negative patients (22.4 Versus 8.9ng/ml). Thus, the serum SCCa had a statistically significant association with lymph node metastasis.

Besides what we mentioned above, there are a series of article studied the role of pretreatment squamous cell carcinoma antigen in predicting nodal metastasis in cervical cancer from which we could find the true positivity, true negativity, false positivity and false negativity at the very SCC cut-off vale it used. Xiong[20] in 2009 studied 100 patients staging IB1-IVB at SCCa 1.5ng/ml. Gaarenstroom[11] in 1995 investigated 78 stage IA-IVA patients using SCCa 1.5ng/ml. DUK[13] in 1996 studied 653 patients of stage IB-IV with SCCa 1.9ng/ml. Li[21] in 2015 studied 282 patients IIB-IV with SCCa 3.5 ng/ml. Wei[22] studied 127 patients I-II with SCC 1.5ng/ml. Takeda[23] in 2002 studied 103 patients IB-IIB with SCCa 1.5ng/ml. Li Q[24]in 2015 studied 1394 patients I-IIA with SCCa 2.0ng/ml. Luan[16] in 2012 studied 365 patients staging 0-IV using SCCa at a series of 1.5,2 and 4.5ng/ml. Van de Lande[25] in 2009 studied 91 patients staging IB2-IIA. Scambia[26] in 1994 studied 102 patients staging I-IV using SCCa at a series of 2.5, 5 and 7ng/ml. The results of those researches mentioned above were listed in Table 1.The general characteristics of 13 articles included for the diagnostic meta-analysis. There were 17 sets of data (TP,TN,FP and FN) from 13 articles [11, 13, 16, 20–29]which were summarized in Table 1. The quality evaluation via QUADAS of 13 studies was demonstrated on Table 2.

**Table 1:** The general characteristics of the including studies.

Study	Year	Patient	Lymph Node	TP	FP	FN	TN	Sensitivity (%)	Specificity (%)	PPV(%)	NPV(%)	SCC Value	Stage	QUADAS value
Bae	1997	67	67	10	9	5	43	66.67	82.69	52.63	89.58	2.0	IB2-IIB	26
Duk	1996	653	252	42	59	23	128	64.62	68.45	41.58	84.77	1.9	IB-IV	23
Gao	2010	110	110	17	9	10	74	62.96	89.16	65.38	88.10	4.0	IB-IIA	26
Gaarenstroom	1995	78	33	5	10	4	14	55.56	58.33	33.33	77.78	1.5	IA-IVA	24
Li	2015	1394	685	80	398	24	183	76.92	31.50	16.74	88.41	2.0	I-IIA	26
Li	2015	286	220	34	62	8	116	80.95	65.17	35.42	93.55	3.5	IB1-IIIB	26
Luan(i)	2012	365	365	50	144	16	155	75.76	51.84	25.77	35.42	1.5	0-IV	19
Luan(ii)	2012	365	365	47	98	19	201	71.21	67.22	32.41	91.36	2.0	0-IV	19
Luan(iii)	2012	365	365	32	54	34	245	48.48	81.94	37.21	87.81	4.0	0-IV	19
Scambia(i)	1994	102	74	13	35	2	24	86.67	40.68	27.08	92.31	2.5	IB-IV	20
Scambia(ii)	1994	102	74	10	20	5	39	66.67	66.10	33.33	88.64	5.0	IB-IV	20
Scambia(iii)	1994	102	74	8	14	7	45	53.33	76.27	36.36	86.54	7.0	IB-IV	20
Takeda	2002	103	103	22	33	6	42	78.57	56.00	40.00	87.50	1.5	IB-IIB	26
Van	2009	91	91	14	15	11	51	56.00	77.27	48.28	82.26	1.7	IB2-IIA	26
Wei	2014	127	127	47	62	2	16	95.92	20.51	43.12	88.89	1.5	I-II	24
Xiong	2009	100	77	18	15	6	38	75.00	71.70	54.55	86.36	1.5	IB1-IVB	24

**Table 2:** The results of quality evaluation of QUADAS.

Study	Total	Quality indicators From Quadas Scale													
		1	2	3	4	5	6	7	8	9	10	11	12	13	14
Van	26	2	2	2	2	2	2	2	2	2	1	1	2	2	2
Li	26	2	2	2	2	2	2	2	2	2	1	1	2	2	2
Luan	19	0	0	1	2	2	2	2	2	2	1	1	2	2	0
Scambia	20	0	0	2	2	2	2	2	2	2	1	1	2	2	0
Li	26	2	2	2	2	2	2	2	2	2	1	1	2	2	2
Huang	24	0	2	2	2	2	2	2	2	2	1	1	2	2	2
Gaarenstroom	24	2	0	2	2	2	2	2	2	2	1	1	2	2	2
Duk	23	2	0	2	2	2	2	2	2	2	1	1	2	2	1
Wei	24	0	2	2	2	2	2	2	2	2	1	1	2	2	2
Xiong	24	2	0	2	2	2	2	2	2	2	1	1	2	2	2
Takeda	26	2	2	2	2	2	2	2	2	2	1	1	2	2	2
Takeshima	22	2	2	2	2	2	2	2	2	2	1	1	2	0	0
Gao	26	2	2	2	2	2	2	2	2	2	1	1	2	2	2
kim	28	2	2	2	2	2	2	2	2	2	2	2	2	2	2
Bae	26	2	2	2	2	2	2	2	2	2	1	1	2	2	2

Score 2 means yes, 1 means not clearly and 0 means no.

For this study, there are 14 items to evaluate the studies. 1.Were the patients all cervical squamous cancer? 2.Were selection criteria clearly described? 3.Were pathological diagnosis reference standard? 4.Did the patients have SCCa evaluation before treatment? 5.Have all the patients have lymph node pathological diagnosis? 6.Was pathological diagnosis the only standard for lymph node metastasis? 7.Were the pathological diagnosis independent of the SCCa test? 8.Can SCCa be repeated? 9Was the execution of pathological diagnosis described sufficiently enough to permit replication? 10Was the SCCa interpreted without knowledge of the pathological results? 11.Was the pathological results interpreted without the knowledge of the SCCa? 12.Were the clinical data credible? 13.Did they report all the results including the cases difficult to explain? 14.Were withdrawals from the study explained?

### Heterogeneity test

**Fig 2:**
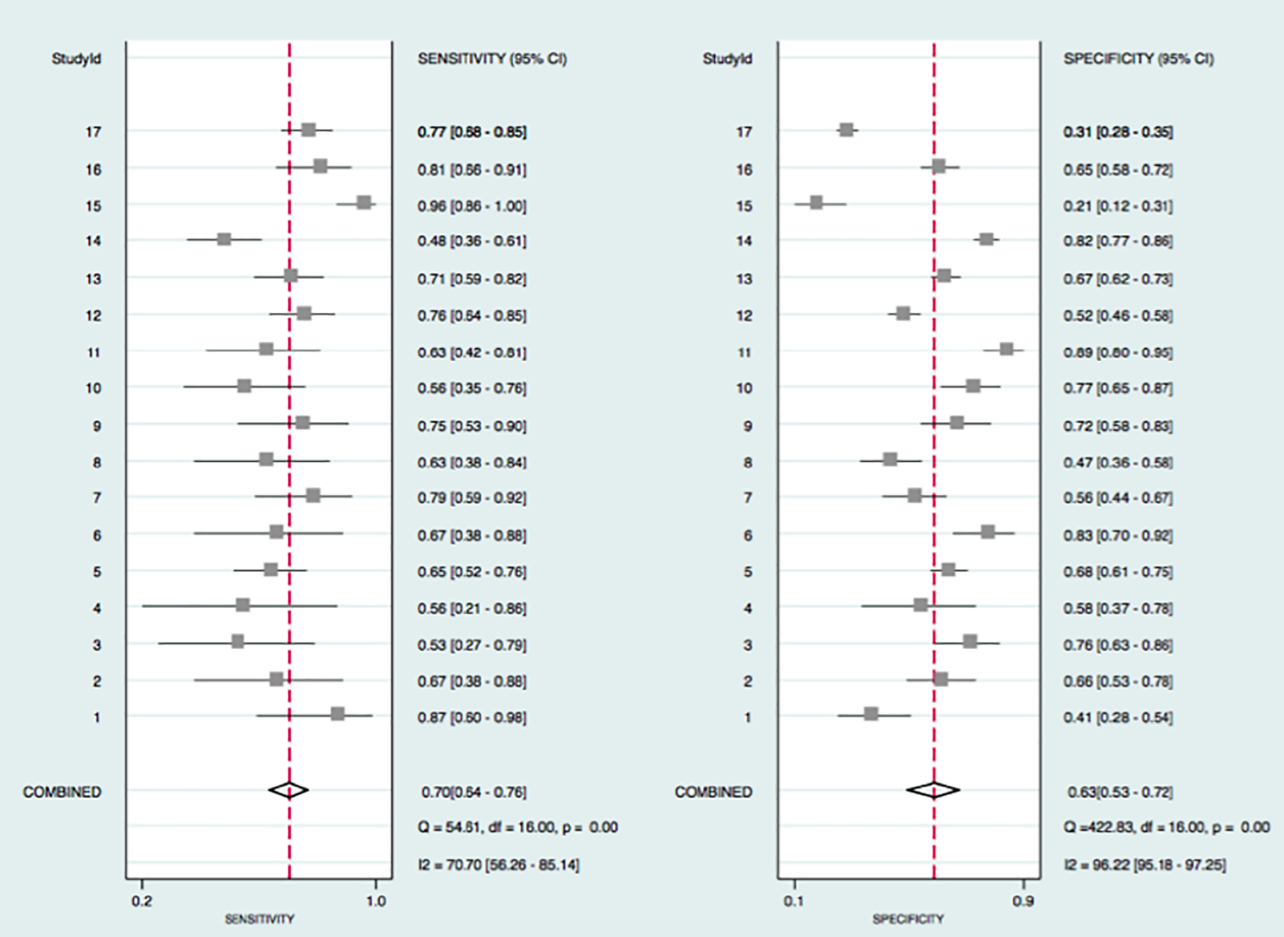
The forest plot about the pooled sensitivity and specificity for the diagnostic value of SCCa on the lymph node metastasis was drawn. And the heterogeneity could be determined on the figure. The Irawn. And the heterogeneity could be determined on the figure. SCindicating there were heterogeneity between studies.(I^2^>50 suggested heterogeneity existed). The pooled sensitivity was 0.7 and specificity was 0.63, indicating a medium diagnostic power.

**Fig 3:**
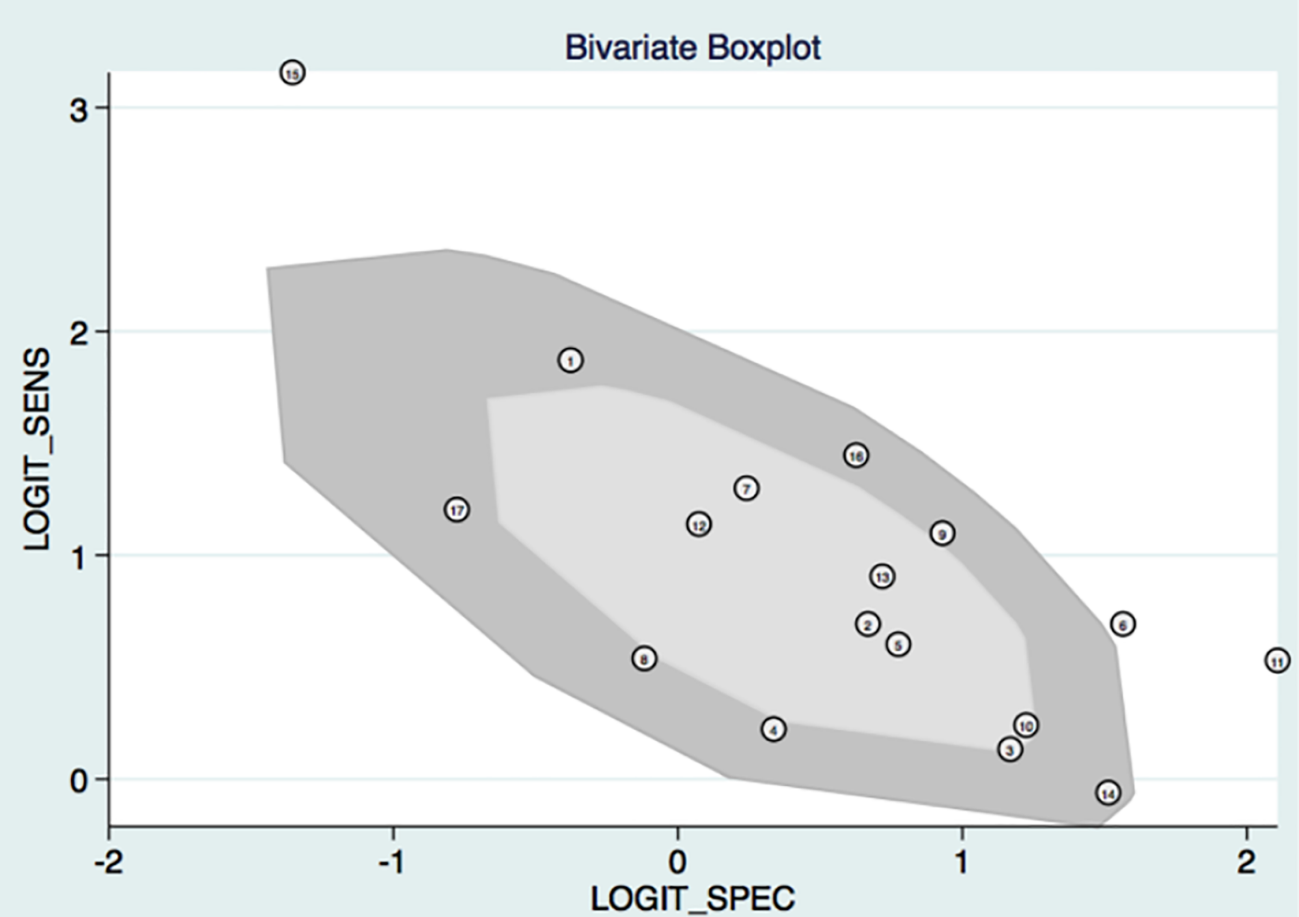
The bivariate boxplot about the heterogeneity was drawn. It demonstrated that 3 sets of data were out of the circles, which indicated there was heterogeneity between articles we included.

The proportion of heterogeneity likely due to threshold effect was 0.91 by Stata 14.0, which indicated there was threshold effect. As can be seen in Fig 2, I^2^ for sensitivity and specificity was 70.7 and 96.2. *P*<0.05. Besides, the bivariate boxplot (Fig 3) showed 3 articles were out of the circles indicating there was heterogeneity between articles we included. Then we utilized the random effect model in the following meta-analysis. The heterogeneity was significant which call for subgroup analysis. We also carried out a subgroup analysis and meta-regression based on year, quality score FIGO stage and SCC.

### Synchronizing SROC

**Fig 4:**
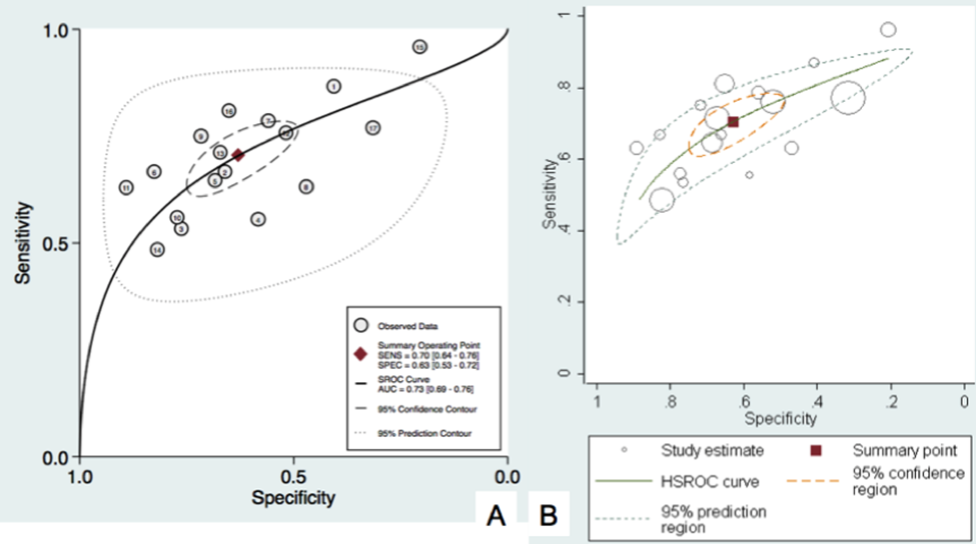
SROC curve was drawn in this meta-analysis. The random effect model was used via bivariate model (Fig 4A) and HSROC model (Fig 4B). The pooled sensitivity and specificity were 0.70 and 0.63 respectively. AUC was 0.73 indicating a medium diagnostic value.

Traditional fixed effect model was not applicable for our analysis considering there was heterogeneity. In order to calculate the SROC curve, two methods were feasible. One was to use the bivariate model in STATA software. And another solution was to utilize HSROC model.They could both calculate the data with heterogeneity even though they based on different mathematical theories(Fig 4). But the HSROC model was not widely used by statistician and HSROC model could not provide the AUC value which reflected the diagnostic power. We decided to use both of them to draw the SROC curve and to calculate the AUC(Fig 4A and 4B).

### Subgroup analysis

We subdivided the articles by SCCa levels, which were <1.5,1.5–2,2–3 and >3ng/ml. The I subdivided the articles by and *P* in each subgroup were larger than 0.05, which indicated the heterogeneity was partially from the SCCa cut off value. Then we conducted meta-regression analysis with publication year, quality evaluation of including studies and FIGO stage. No significant relativity was found.

### Publication bias

**Fig 5:**
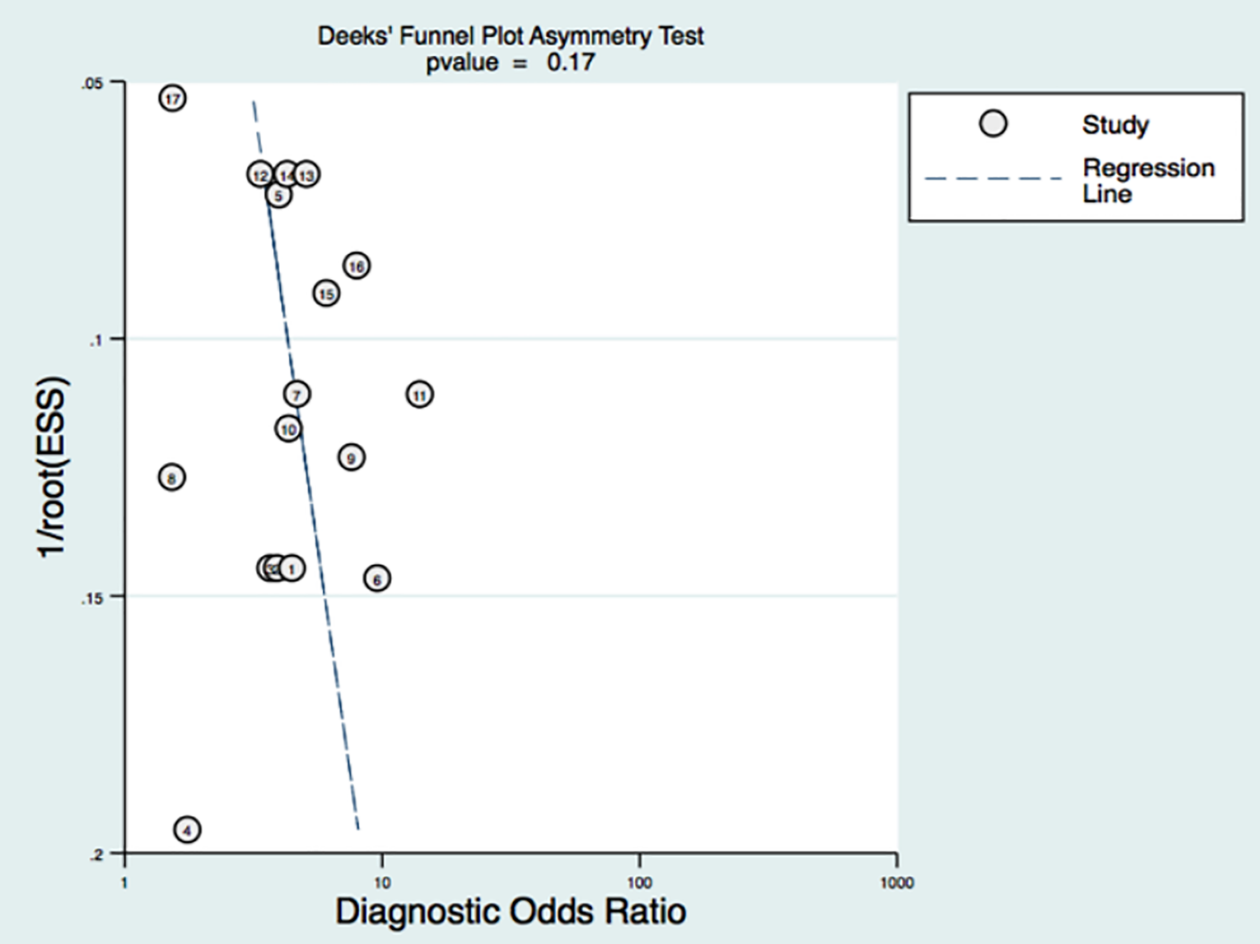
The funnel plot about the publication bias was drawn. As can be seen no asymmetry was found in the funnel plot, indicating low likelihood of publication bias.

Random error and bias affect the diagnostic value of a test. The narrower the 95%CI, the more accurate the test would be. Publication bias could be reflected by the funnel graph. In Fig 5, no funnel plot asymmetry was found for the association between lymph node metastasis and an incremental increase in SCCa. *P* value was 0.17, which indicated a low probability of publication bias.

## Discussion

Even though there were numerous reports in the literature regarding the relationship of SCCa and lymph node status, no consensus had been reached. We conducted this meta-analysis in order to decide the value of SCCa on lymph node metastasis on a large scale. Thus, we strictly followed the inclusion and exclusion criteria to guarantee the quality of including articles and utilized the Quadas scale to insure the articles were of relatively high quality. 13 articles, 17 sets of data and 3985 patients were included for this meta-analysis. The funnel plot analysis indicated no publication bias was found. The forest plot and bivariate box plot showed there was heterogeneity between articles. Subgroup analysis and meta-regression analysis suggested the heterogeneity was from SCC cut off value. The random effect model was utilized to meta-analyze the diagnostic value of SCC.

### Predictive value of SCCa on lymph node status

According to statistics theory, HSROC model and bivariate model could both calculate the data with heterogeneity via random effect model. The HSROC model based on a different mathematical theory called bayes theory. Compared with bivariate model, HSROC was briefer. But AUC value was not available in the HSROC. In order to calculate the SROC curve accurately, we used both of them. The curves were similar. From the SROC curve we obtain the pooled sensitivity was 0.70 and specificity was 0.63. AUC was 0.73.

Besides, there are 8 articles which investigated the relative risk of lymphatic metastasis on SCCa elevation. For instance, Strauss in 2001 studied 129 patients of stages IA2–IIB and found patients with SCC >3.0ng/ml had a high risk of positive pelvic nodal status (Risk Ratio = 14.0).Similar outcomes were showed in other 7 articles which could be seen in Table 3. Interestingly, We noticed that compared with other studies, the OR value in Takeshima’s article seemed strikingly high (40 folds) and nearly 2/3 of patients have pelvic lymph node metastasis when SCCa levels exceed 4ng/ml.[30] We tapped into the underlying factors accounting for that. We found unlike other studies which included patients from I to IV stage, Takeshima exclusively enrolled stage IB patients. Since SCCa values reflected not only the lymph node status but also the tumor volume and FIGO stages, the relative significance between lymph node and SCC was supposed to be much stronger and significant when excluding the impact of FIGO staging on the SCC level.

**Table 3:** The articles which provided the information about the relationship between the elevated SCCa and the risk of pelvic nodal metastasis.

Study	Year	Number	SCCa (ng/ml)	Risk Ratio	FIGO Stage	Site of lymph node metastasis
Takeda	2002	103	>1.5	3.7(1.3–10.7)	IB	Pelvic lymph node
Zhao	2016	237	>2.2	2.45(1.15–5.24)	IB1-2B	Pelvic lymph node
Strauss	2002	129	>3	14	I -IIB	Pelvic lymph node
Huang	2010	960	>4	2.3(1.1–4.8)	IB -IIB	Common iliac lymph node
Huang(i)	2012	188	10–40	4.2(1.7–10.6)	I-IV	PALN metastasis
Huang(ii)	2012	188	>40.0	12.6(4.8–32.7)	I-IV	PALN metastasis
Ogino	2005	99	>10.8	6.1(2.0–18.5)	IIB-IVA	PALN metastasis
Feng	2005	205	>4.0	4.2	I-IIB	Pelvic lymph node
Takeshima	1998	136	>4.0	40.0	IB	Pelvic lymph node

For this part we concluded that even though the sensitivity, specificity and AUC were not that satisfactory, the patient with SCCa elevation had higher risk of lymph node metastasis than the one with normal SCCa. From the results we are convinced that SCCa could be a good parameter to assist the diagnosis of lymphatic metastasis together with CT or MRI.

### Predictive value of SCCa on para-aortic lymph node metastasis

Para-aortic lymph node (PALN) recurrence is one of the distant recurrences, which is often associated with other simultaneous metastatic lesions and usually shows a poor outcome[31]. PALN recurrence after treatment is not unusual. It has a high mortality within 2 years despite aggressive salvage treatment[32]. The prediction of isolated para-aortic node recurrence significantly correlated with SCCa elevation as the initial sign has been reported previously [17, 31, 33]. Chou found SCCa>4ng/mL was a statistically significant predictive factor for para-aortic lymph node recurrence. Huang et al found the SCCa of 10–40 and >40ng/ml had a high risk for PALN recurrence with Hazard Ratio (HR) of 4.24 and 12.55 respectively. Ogino demonstrated SCCa >10.8ng/ml increased the risk of para-aortic lymph nodal metastasis by 6.06 folds. Those researches concluded SCCa was an important risk factor for PALN recurrence. For patients with SCCa elevation, PET-CT examination, laparoscopic extraperitoneal paraaortic lymphadenectomy or prophylactic PALN irradiation for radiotherapy planning should be considered first.

It used to be reported a 100% mortality rate within 2 years after isolated PALN recurrence[32]. But in another study including 876 cervical cancer patients, the 5 year survival rate was 30.8%[31]. We drilled down this study and found that more than half patients in this study had no clinical symptom and sign, and the PALN recurrence was diagnosed by an elevated value during routine tumor marker check-up and an abnormal finding at a subsequent image study and biopsy. Thus, the survival benefit would be due to early detection of isolated PALN recurrence, which was initially identified by an elevated value for tumor markers during regular follow-up. Chou declared that PALN recurrence after primary irradiation is a curable disease if diagnosed early. On the contrary, Lekskul [34]affirmed SCCa level was significantly related to para-aortic lymph node status, but he did not think SCCa was a good predictor for pelvic and para-aortic lymph node metastasis because the area under ROC was 0.59 (Usually the area<0.70 was recognized as low diagnostic efficiency). We tapped into this study and found the patients with cervical cancer from stage IB2 to IVA were included, consequently with variable tumor burden. So the effects of tumor size on SCCa were unavoidable, which might be the partial reason of low ROC.

To sum up, routine checking of SCCa in all patients and regular imaging studies are helpful for the early diagnosis of PALN recurrence. 10 ng/ml could be potentially considered as a proper cut off value. But the number of articles regarding this is limited, so it still needs further investigation.

Pretreatment SCCa>5ng/ml was subgroup of high-risk node-positive patients in relatively early stage cervical cancers (stage IB and IIA). For patients in this high-risk category, 50% risk of tumor recurrence would occur after surgery no matter what adjuvant therapy was given. So radical hysterectomy should not be attempted if the pretreatment SCCa level was above 5ng/ml[35].Women with SCCa higher than 2ng/ml are almost five times more likely than women with a normal SCCa to receive adjuvant treatment[3].

Of course there is limitation in our study. Heterogeneity existed between studies. We partially ascribe this to the SCCa threshold. But we are not determined the other factors contributing to the heterogeneity.

In conclusion, this meta-analysis confirms the prediction value of SCCa even though the predictive value was medium. Elevated SCCa might still provide meaningful information, in additional to CT or MRI, to the surgeon. There are still some potential benefits as it is correlated with para-aortic lymph node metastasis. Consideration must be given to the possibility of altering treatment for patients with elevated SCCa. These patients might be potential candidates for laparoscopic assessment of pelvic or/and para-aortic lymph nodes. Identifying them is of clinical value because it allows more accurate preoperative counseling in relation to the adjustment of the treatment strategy.

## Supporting information

S1 Checklist PRISMA checklist. (DOC)
