# Supplementary material for: The value of squamous cell carcinoma antigen (SCCa) to determine the lymph nodal metastasis in cervical cancer: A meta-analysis and literature review
Source: PLoS One. 2017 Dec 11;12(12):e0186165. doi: 10.1371/journal.pone.0186165 (PMC5724822; doi:10.1371/journal.pone.0186165)
Supplement: S1 Checklist — (DOC) [file pone.0186165.s001.doc]

| **Section/topic** | **#** | **Checklist item** | **Reported on page #** |
| --- | --- | --- | --- |
| **TITLE** | | |  |
| Title | 1 | The value of Squamous cell carcinoma antigen (SCCa) to determine the lymph nodal metastasis in cervical cancer: A Meta analysis and literature review | 1 |
| **ABSTRACT** | | |  |
| Structured summary | 2 | **Background:** The diagnostic power of CT or MRI on the lymph node status was limited. Supplement measurements were needed to assist the diagnosis of lymph node metastasis. The SCCa was reported to be close related to lymph node status. But currently the clinical value of serum SCCa measurement in lymph node status has not been clearly defined. This meta-analysis was to investigate this topic on a large scale.  **Method**: Searching the Pubmed, Embase, Cochrane library, CNKI and Wanfang database for SCC-Ag/SCCA/SCC-antigen and cervical cancer/tumor/carcinoma/neoplasm. QUADAS (quality assessment of diagnostic accuracy studies) was used to evaluate the quality of the articles. The 4 space table data were extracted and analyzed by STATA 14.0. The forest plot and bivariate boxplot were utilized to evaluate the heterogeneity. The funnel graph was used to test the publication bias. The SROC curve was draw via random effect model and HSROC model.  **Result:** 17 sets of 4-space table data and 3985 patients were included for the diagnostic meta-analysis. There was heterogeneity, which was partially from SCCa cut-off value. The pooled sensitivity was 0.70 and specificity was 0.63. AUC was 0.73. Eight articles provided the RR/HR/OR of lymphatic metastasis when SCCa increased. The pooled RR was 4.11, which meant the patients with elevated SCCa were at risk of lymph node metastasis 4 times higher than the one who has normal SCCa level.  **Conclusion:** Even though SCCa was not that satisfactory predictor for lymph nodal metastasis, it was strongly related to lymph node status. SCCa could assist imaging tests to detect lymph node metastasis. Besides, it was correlated with para-aortic lymph node metastasis. | 1-2 |
| **INTRODUCTION** | | |  |
| Rationale | 3 | Squamous cell carcinoma antigen (SCCa) is the most widely used and most reliable tumor marker for squamous cell carcinoma | 3 |
| Objectives | 4 | We went over these studies about the experience of SCCa elevation in relation to lymph node status and then meta-analyzed them on a large scale, in order to determine whether SCCa can predict the presence of nodal metastasis. | 3 |
| **METHODS** | | |  |
| Protocol and registration | 5 | We strictly follow the protocol to select articles, extract data and analyze data. | 3-4 |
| Eligibility criteria | 6 | Studies were included if they fulfilled the following criteria:(1) the cases which were diagnosed with cervical carcinoma (2) in the case group the lymph node metastasis was confirmed by pathology (3) the SCCa test was certain and could be repeated; (4) the results was presented by four space form (5) The RR/OR/HR, the number of cases, FIGO stage, must be also provided (or data to calculate these). When there were multiple publications from the same population, the study that included the most recently updated data was included. Excluded if 1) No four space form or RR/OR/HR was provided. 2) The results were incompleted. 3) Lymph nodal metastasis was not confirmed by pathology. | 3-4 |
| Information sources | 7 | We search the Pubmed, Embase, Cochrane library,CNKI and Wanfang database for pertinent articles published in any language from1990 to 2017 and by manually searching the reference list of the computer retrieved publications | 3-4 |
| Search | 8 | For the computer searches we used the following MeSH terms or text words: A or a combined with B or b. A was defined as squamous cell carcinoma antigen, SCC-Ag, SCCA, SCC-antigen. B was defined as cervical cancer/tumor/carcinoma/neoplasm. | 4 |
| Study selection | 9 | We selected articles related with the SCCa and cervical cancer by title. Then we read the abstract to pick out articles associated with SCCa and lymph node status. Then we read the full text and pick out articles which were highly related to our topic | 4 |
| Data collection process | 10 | Two authors independently completed the data extraction. Any disagreements were resolved by discussion and end with consensus | 4 |
| Data items | 11 | Year, patient number, true positive, true negative, false positive,false negative,sensitivity,specificity SCCa  Value,FIGO stage | 4 |
| Risk of bias in individual studies | 12 | We evaluate the bias according to the Agency for Healthcare Rearch and Quality(AHRQ) evidence based practice center(EPC) methods guide | 4-5 |
| Summary measures | 13 | true positive, true negative, false positive,false negative | 5 |
| Synthesis of results | 14 | Pooled sensitivity，specificity，AUC | 5 |

Page 1 of 2

| **Section/topic** | **#** | **Checklist item** | **Reported on page #** |
| --- | --- | --- | --- |
| Risk of bias across studies | 15 | We used the funnel plot to evaluate the publication bias | 4-5 |
| Additional analyses | 16 | subgroup analyses was done | 5 |
| **RESULTS** | | |  |
| Study selection | 17 | We found 142 articles are related with the SCCa and cervical cancer by title. Then we read the abstract one bye one and we found 42 articles were associated with SCCa and lymph node status. Then we read the full text of them, and pick out 17 articles which were highly related to our topic and 4 articles were excluded after quality evaluation. Finally 13 articles were included. | 5-6 |
| Study characteristics | 18 | We have done the summary of each article in table 1 | 17 |
| Risk of bias within studies | 19 | No bias was found | 9 |
| Results of individual studies | 20 | We have done the summary of each article in table 1 | 17 |
| Synthesis of results | 21 | The pooled sensitivity was 0.70 and specificity was 0.63. AUC was 0.73. Eight articles provided the RR/HR/OR of lymphatic metastasis when SCCa increased. The pooled RR was 4.1 | 8-9 |
| Risk of bias across studies | 22 | No publication bias was found | 12-13 |
| Additional analysis | 23 | We have provided the results of subgrouping in the manuscript | 9 |
| **DISCUSSION** | | |  |
| Summary of evidence | 24 | 1)Even though the sensitivity, specificity and AUC were not that satisfactory, the patient with SCCa elevation still had about 4 times higher risk (RR=4.11) of lymph node metastasis than the one with normal SCCa.  2) Routine checking of SCCa in all patients and regular imaging studies are helpful for the early diagnosis of PALN recurrence. 10 ng/ml could be potentially considered as a proper cut off value. But the number of articles regarding this is limited, so it still needs further investigation. | 10 |
| Limitations | 25 | There is heterogeneity between studies and partially due to the SCCa threshold. But we are not determined the other factors contributing to the heterogeneity. | 13 |
| Conclusions | 26 | SCCa was strongly related to lymph node status, thus it could assist imaging tests to detect lymph node metastasis. Besides, it was correlated with para-aortic lymph node metastasis. | 13 |
| **FUNDING** | | |  |
| Funding | 27 | No |  |

*From:*  Moher D, Liberati A, Tetzlaff J, Altman DG, The PRISMA Group (2009). Preferred Reporting Items for Systematic Reviews and Meta-Analyses: The PRISMA Statement. PLoS Med 6(7): e1000097. doi:10.1371/journal.pmed1000097

For more information, visit: **www.prisma-statement.org**.

Page 2 of 2
